# The Effect of Physician-Led Enhanced Care Teams in Prehospital Trauma Resuscitation

**DOI:** 10.7759/cureus.10405

**Published:** 2020-09-12

**Authors:** Clayton Chiapuzio, Thomas Dang, Shannon Meagher, Brandon Woodward, Michael Neeki

**Affiliations:** 1 Emergency Medicine, Arrowhead Regional Medical Center, Colton, USA; 2 Surgery, Arrowhead Regional Medical Center, Colton, USA; 3 Emergency Medicine, California University of Science and Medicine, Colton, USA

**Keywords:** major trauma, hemorrhagic shock, enhanced care teams, emergency medical service, prehospital emergency medicine

## Abstract

Trauma is the leading cause of morbidity and mortality for those under 45 years of age in the United States with half of the deaths in trauma being attributed to hemorrhagic shock. The use of enhanced care teams (ECTs) that include physicians in selective prehospital settings has allowed the delivery of advanced critical care interventions in the field. We present a unique case where a young driver involved in a motor vehicle accident was trapped under the weight of his vehicle, causing extended extrication time. An ECT from the closest trauma center was able to deliver massive transfusion and definitive airway care while the patient was being extricated. While previous literature regarding the benefit of ECTs has been debated, this case suggests a unique niche where rapid deployment of an ECT to the scene made a pronounced difference in survival of the patient.

## Introduction

In the United States (U.S.), non-accidental injury is the third leading cause of death overall, remaining as the leading cause of mortality in those below 45 years of age [1]. Among deaths caused by trauma, approximately half are attributed to hemorrhagic shock. Prompt blood transfusion is known to be a critical component of resuscitation in hemorrhagic shock, but is often not widely provided in a prehospital setting [2]. Despite care being provided, victims of civilian trauma often succumb to their injuries outside the hospital setting.

Over the last decade, advances in civilian trauma care have accelerated due to the integration of experiences from trauma management during the war in Afghanistan [3]. During the war, injured U.S. service members saw a decrease in case fatality rate despite seeing an increase in the severity of injury among U.S. troops during the same time period. These findings have led to the gradual improvements of civilian prehospital care, which is defined as emergency medical care that is given to patients before arrival to a regional trauma center [4].

In different regions across the world, the implementation of trauma care differs depending on the development of the region and resources available. In the majority of regions in the U.S., prehospital trauma care is usually provided by emergency medical service (EMS) personnel, such as emergency medical technicians (EMT) or paramedics [5]. These response teams are capable of providing limited prehospital trauma care [4]. Addition of enhanced care teams (ECTs) incorporates trained physicians to the typical response team. It has allowed the delivery of certain critical care interventions, including prehospital advanced airway management, blood product transfusion, administration of drugs beyond existing prehospital protocols, amputation of entrapped limbs, escharotomy, tube thoracostomy, and pericardiocentesis [6]. These ECTs are capable of performing advanced care that was previously delayed until the patient's arrive at a hospital setting. While the value and cost-effectiveness of this higher tier of care are unclear, ECTs can provide valuable resources in appropriate situations.

Here, we present a case where a victim of a motor vehicle accident was entrapped for a prolonged amount of time, and an appropriate ECT deployment in coordination with a local prehospital EMS team provided the early stages of trauma resuscitation in a prehospital setting.

## Case presentation

Prehospital course

At approximately 0445 on 11/9/2019, a 24-year-old restrained male driver, who allegedly fell asleep traveling on a major highway at high speeds, lost the control of his vehicle, rolled his vehicle over the center divider into oncoming traffic, resulting in a collision with a 29-year-old male driving in the opposite direction. Shortly after, the closest rescue team from the city of Colton Fire Department was able to extract, stabilize, and transport the 29-year-old male to the closest regional trauma center, Arrowhead Regional Medical Center (ARMC). While the trauma team attended to the first victim at the trauma center, another EMS crew was trying to extricate the 24-year-old male from his entangled vehicle.

**Figure 1:**
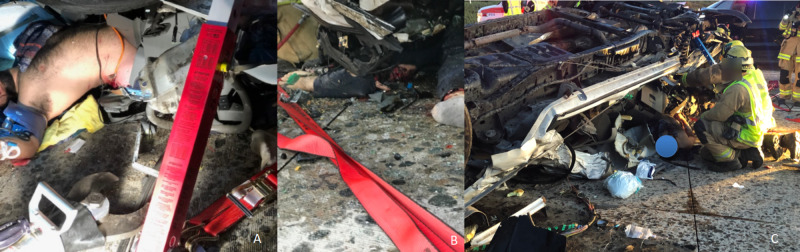
(A) The patient entrapped at level of pelvis and right proximal humerus. (B) The patient’s right lower extremity pinned under vehicle. (C) Colton Fire Department attending to the trapped patient.

Based on the initial evaluation of the scene by the incident commander, a prolonged extrication time was estimated due to the entrapment of the driver and possible need for field amputation of limbs in order to free the patient. Given the estimated prolonged on-site time, the Hospital Emergency Response Team (HERT), a variation of ECT in our region, was activated by the request of the incident commander from the regional dispatch. Due to the after-hour time of the activation, the ECT in this case consisted of a trauma surgeon, emergency medicine resident, and a trauma nurse. After arriving on the scene, the ECT developed the initial plan to perform an advanced airway procedure, apply tourniquets to the mangled limbs, amputate limbs if indicated to extricate the victim, and transport the patient back to the trauma center for further assessment and definitive care. Brief assessment revealed that the patient was trapped at the level of the pelvis and the left proximal humerus under the weight of his entangled vehicle, making field amputation inadvisable (Figure 1).

At the time of the ECT’s arrival, patient’s vitals included blood pressure of 130/74 mmHg, heart rate of 130 beats per minute, and Glasgow Coma Score (GCS) of 15, and the patient was able to speak in full sentences but was in severe distress. Intravenous access was established using a 16-gauge needle in the right antecubital fossa. After the initial assessment, the main concerns for the ECT team remained to be a prolonged extraction time, the patient’s declining ability to protect his airway, continuing hemorrhage from his right lower extremity, and a concern for exsanguination once pressure was relieved from the tamponaded extremities during extraction. The weight of the vehicle surpassed the capability of the local rescue team equipment, and a large capacity crane was requested from the county fire department and local private contractors. This need for additional heavy equipment drastically increased victim entrapment time.

Based on the circumstances and proximity of the trauma center to the scene of the accident, the team initiated massive transfusion protocol from the field with the coordination of ARMC’s base station. During the initial resuscitation, tourniquets were loosely applied to accessible limbs, and four units of packed red blood cells and one gram of tranexamic acid (TXA) were administered. Afterwards, a critical care transport team was requested from the regional ambulance transporting agency. Gradually the patient’s level of consciousness began to decline, and the decision was made to secure his airway utilizing an endotracheal tube with rapid sequence intubation. The patient was then induced with 30 milligrams (mg) intravenous etomidate and 100 mg intravenous rocuronium, followed by intubation using a bougie with an 8.0-millimeter cuffed endotracheal tube (ETT). Given concern for possible ETT dislodgement during the extraction process, the decision was made to place the ETT in the right mainstem bronchus. After successful intubation, the patient was sedated using 5 mg intravenous midazolam and, analgesic control was achieved using 100 micrograms intravenous fentanyl.

An hour after the ECT’s arrival, the heavy crane arrived to lift the vehicle. Once successfully extricated, the patient was secured on a backboard, and double tourniquets were applied on both lower extremities and the left upper extremity. The patient was loaded into the critical care ambulance and was transported to ARMC.

Trauma center resuscitation

**Figure 2:**
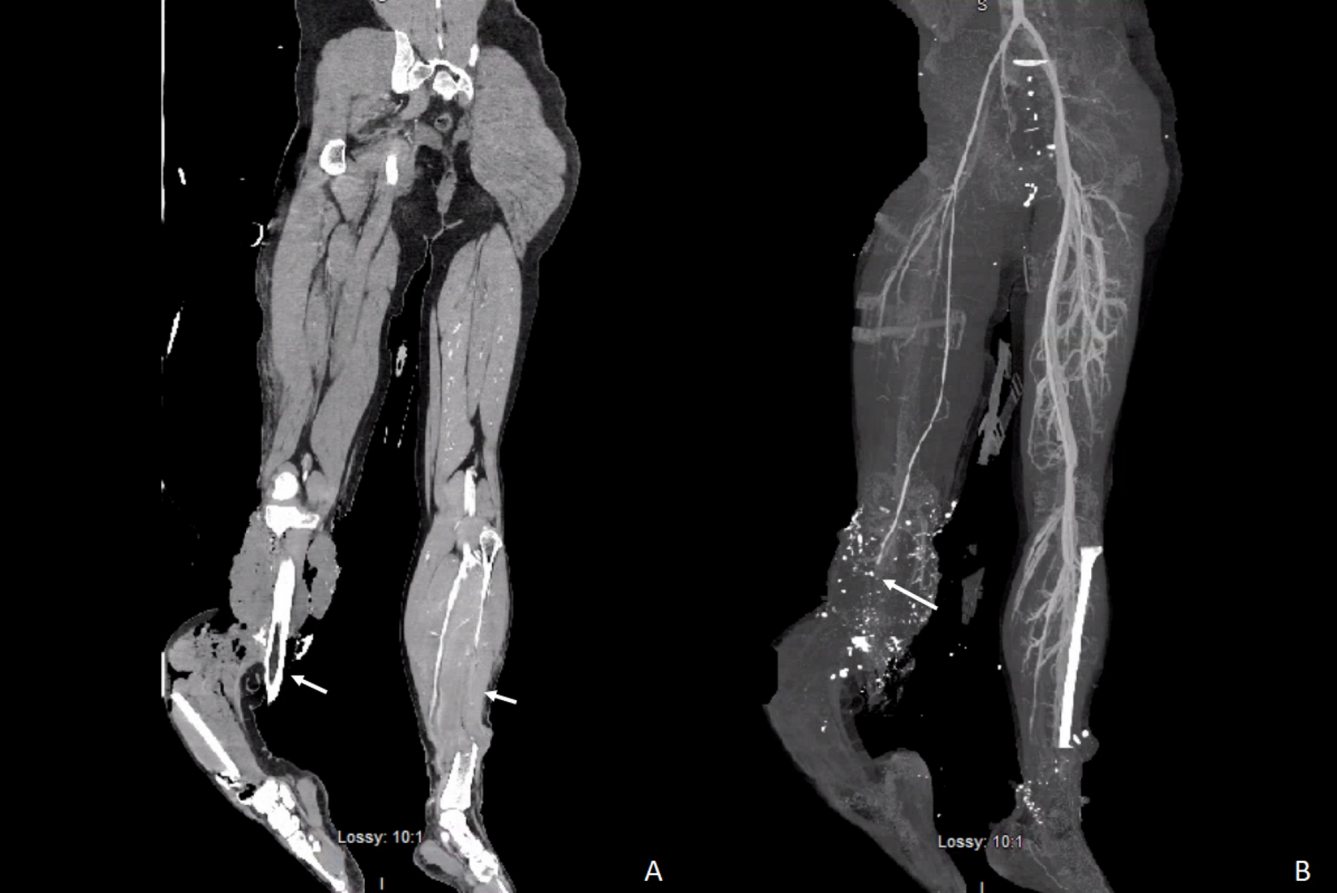
(A) CT of lower extremities without contrast showing bilateral compound fractures of tibia and fibula (white arrows). Right lower extremity was later amputated. (B) Vascular study showing interruption of tibial artery in right lower extremity (white arrow).

The patient arrived in ARMC’s trauma bay approximately two and a half hours after the motor vehicle accident. Upon arrival, the primary survey revealed decreased breath sounds of the left chest with subsequent ultrasound confirming decreased sliding for the left lung. A left chest tube and a left subclavian cordis line were placed. It was also noted that the right lower extremity below the knee sustained severe crush injury and would require surgical amputation. Repeat vital signs were noted to be improving, and the patient underwent imaging with CT. A CT scan of the chest, abdomen, and pelvis revealed a mild right lung contusion and right comminuted inferior pubic ramus fracture with minimal diastasis of the sacroiliac joint. There was no evidence of gross organ injury of the chest, abdomen, or pelvis and no evidence of spinal injuries. A CT of the lower extremities with an accompanying vascular study revealed bilateral compound fractures of the tibia and fibula with complete transection of the right anterior tibial artery and tibial peroneal trunk (Figure 2). The patient was taken to the operating room within 30 minutes of arrival by both the trauma and orthopedic surgical teams for the management of the right lower extremity.

Hospital course

On initial surgical intervention, an above the knee (ATK) amputation of the right lower extremity was performed, and external fixation of the left distal tibia was placed. On hospital day 2, the patient’s total creatine kinase, secondary to his prolonged entrapment, peaked at 7,000 units/liter (normal 22-198). On hospital day 3, irrigation and debridement of the left lower extremity and revision of the right ATK amputation were performed. On hospital day 5, the patient had a repeat operating room washout of the left lower extremity. Due to insurance preferences, the patient was transferred to another medical facility on hospital day 10. At one-month follow-up, the patient admitted to continuing palsy secondary to brachial plexus injury of the left hand. He was in the process of obtaining a right lower extremity prosthesis and was three weeks postoperative of the internal fixation of the left lower extremity. He had been working with physical and occupational therapy and was able to return home to his family.

## Discussion

Motor vehicle accidents often result in severe trauma, which places the victim at high risk for death due to airway complications, chest injuries, and hemorrhagic shock [7]. Optimal management includes transporting the patient to the nearest trauma center as quickly and safely as possible for definitive trauma care. While timeliness is a critical factor, there are many considerations that can delay transport, including the distance from the trauma center, the safety of the site, and whether the patient is in a situation that allows them to be transported. In situations where transportation is delayed and limited capabilities are noted by an incident commander, utilization of an ECT may have an important role in the patient's survival.

While an ECT may provide advanced resuscitation, the degree of resuscitation to be performed in a prehospital setting is controversial. Prehospital blood transfusion is generally considered acceptable and is supported by a previous study of U.S. soldiers in Afghanistan that associated the time to initial transfusion with a decreased 24-hour mortality [8]. While blood transfusion is relatively quick, there are concerns that advanced airway management may extend on-site time, which could result in higher mortality in patients undergoing hemorrhagic shock [9]. However, Hoffman et al. discovered that prehospital intubation in patients with a GCS of <8 was associated with better outcomes than in non-intubated patients [10]. Conversely, Schauer et al. discovered that prehospital intubation in military trauma cases was associated with lower survival than those intubated in an emergency department. This may be due to the fact that those requiring in-field intubation generally have suffered more grave injuries [11]. Transport time is a critical factor in trauma cases, and Cowley et al. associated ECTs in cases of penetrating trauma with decreased on-site time overall, which may support the use of ECTs even when transport time is a concern. However, the study is limited by a small cohort size and a lack of analysis of the amount of interventions given [12]. Rapid transport to a trauma center is the optimal goal, but survival after severe trauma depends on not only short rescue time but also well-used rescue time [13]. For our case, expedient transportation of the victim was not possible, so judgment to intubate the patient was made based on his declining condition and the extended extrication time. The presence of an ECT allowed advanced and potentially life-saving resuscitation before definite transport was possible.

The benefits of ECTs for prehospital resuscitation have been widely debated, as emergency response teams in the U.S. generally deploy without accompanying physicians [14]. Researchers have attempted to assess whether there were a significant mortality benefit from ECT over non-physician-assisted response teams, especially when considering that readily available ECTs require more resources [15]. Two systematic reviews associated increased survival in trauma cases and cardiac arrest with prehospital treatment from ECTs, although the evidence was limited, as few studies satisfied each review’s inclusion criteria [16,17]. While literature about ECTs in the U.S. is scarce, studies in European countries have found evidence in favor of physician-assisted response teams. Maddock et al. discovered in Scotland that physician-led prehospital critical care teams (PHCCTs) resulted in reduced mortality in trauma patients, even when considering that patients who required PHCCTs generally had higher injury severity scores (ISS) [7]. Smith et al. performed a study in the United Kingdom and were the first able to discern a risk-adjusted mortality benefit in patients treated by ECTs with no increase in morbidity [18]. On the other hand, Hepple et al. reviewed the United Kingdom’s Trauma Audit and Research Network (TARN) and could not demonstrate an improved adjusted survival rate for patients who were treated by a physician-led ECT [6]. Similarly, Bieler et al. performed a matched-pair analysis of the trauma registry of the German Trauma Society and could not find a significant difference in mortality within the first 24 hours in injury or during hospitalization for patients treated by an ECT [19]. However, the analysis discovered that there was a significant increase in the number of prehospital procedures performed without a significant difference in prehospital time. Bieler et al.’s findings were also supported by a Japan study [20].

While many of these studies were unable to associate ECTs with reduced mortality, one reason for this perceived lack of benefit is that ECTs are generally deployed to more severe cases with victims often presenting with a higher ISS or in a more unstable condition [20]. Each trauma case is unique, and an experienced incident commander should be able to discern whether an ECT is appropriate, especially when resources are limited. Additionally, the populations of interest were usually patients who survived to hospital admission, which creates a blind spot on the effect of ECTs on the subset of patients who do not survive transport to the hospital. Further research in evaluating the effects of ECTs in this subgroup may give insight on the efficacy of physician-assisted response teams in preventing mortality in the prehospital setting.

## Conclusions

In the U.S, ECTs are uncommonly deployed to scenarios of massive trauma and allow advanced prehospital resuscitation. In situations where transportation to a trauma center is delayed, a trained on-site physician may be able to make a real-time difference in survival of victims prior to definite care at a trauma center. Further research into the benefit of ECTs in situations with prolonged entrapment and transportation time may support increased usage of physician-supported response teams.
